# Choosing a cerebral near-infrared spectroscopy system for use in traumatic brain injury: deriving the ideal source detector layout

**DOI:** 10.1186/cc13658

**Published:** 2014-03-17

**Authors:** D Davies, M Clancy, Z Su, H Denghani, A Belli

**Affiliations:** 1 National Institute for Health Research Surgical Reconstruction and Microbiology, Edgbaston, Birmingham, UK; 2 University of Birmingham - School of Computational Science, Birmingham, UK

## Introduction

Cerebral near-infrared spectroscopy (NIRS) represents an exciting prospect for the non-invasive monitoring of cerebral tissue oxygenation in traumatic brain injury (TBI). Earlier attempts at clinical application of cerebral NIRS demonstrated that further work was needed [1]. The basic layout of the probe portion of these devices consists of a light source and a light detector, arranged at calculated distances and configurations in order to observe target tissue. We aim to determine which commercially available NIRS probe represents the most sensitive layout of sources/detectors for the greatest sensitivity in observing tissue oxygenation at the optimal depth for TBI.

## Methods

The optimal depth for grey matter target tissue (grey-white matter junction) was ascertained by reviewing a series of brain CT scans of patients who had sustained a TBI. Set (average) measurements were derived from these identifying the target depth of the grey matter strip from the point of probe placement. Currently there are five commercially available cerebral NIRS systems. Table 1 presents the variety of configurations offered by each device. Source detector layouts were modelled and simulated using the NIRFAST^® ^computational light modelling software (developed at the University of Birmingham) [2]. The novel approach of this modelling system makes no extrapolated assumptions based on subtraction.

**Table 1:** Available source detector layouts

Probe	Wavelengths (nm)	Sources	Detectors	Spacing (mm)	Peak depth sensitivity
Optiplex TX (ISS, IL, USA)	690, 830	4	1	30, 35, 40, 45	8
INVOS (Covidien, MA, USA)	730, 81C	1	2	30, 40	8
EQUANOX (Nonin, MN, USA)	730, 810, 880	2	2	20, 40	7
FORE-SIGHT (CAS Medical, CT, USA)	690, 780, 805, 850	1	2	20, 50	10
NIRO-200NX (Hamamatasu, Japan)	735, 810, 850	1	2	19.2, 20	9

## Results

**Figure 1:**
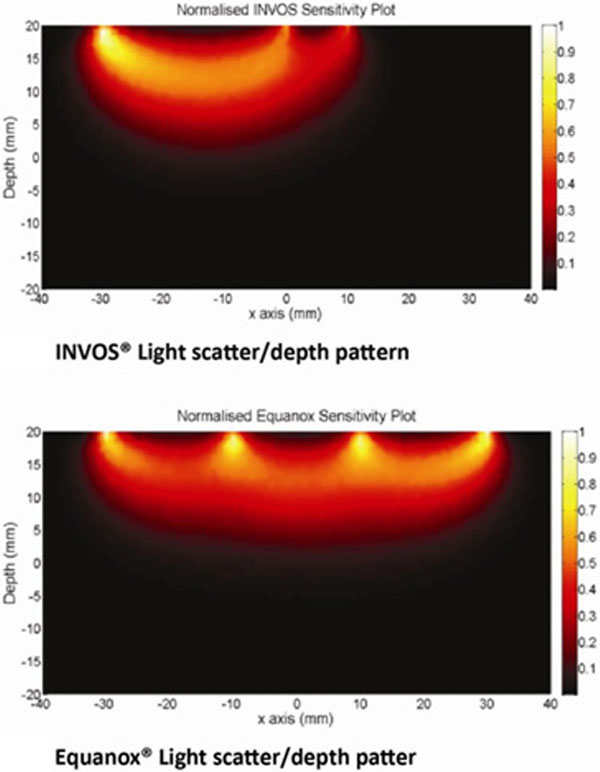
Light scatter predictions of available systems/ illustrated.

**Figure 2:**
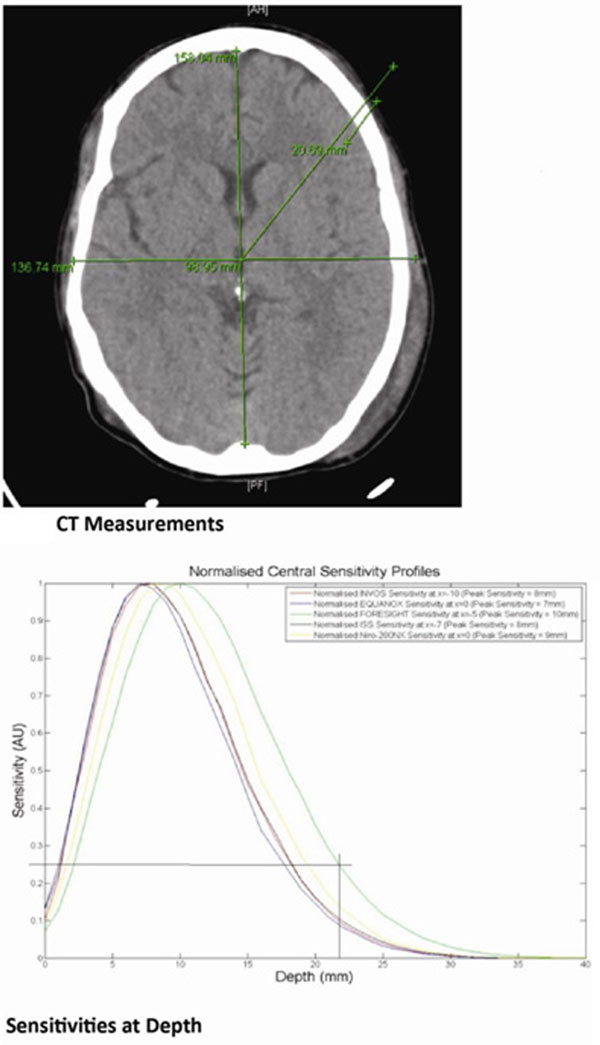
CT measurements and sensitivities at depth

We reviewed 32 trauma series CT brain images and the average depth to grey matter was derived as 21.37 mm (range 16.59 to 31.03 mm, SD 2.33) from the surface (Figure 1). The spectrum of sensitivity of the five probes was modelled (Figure 2). As is apparent here, the FORE-SIGHT (CAS Medical, CT, USA) probe currently offers the greatest sensitivity at our derived target depth.

## Conclusion

Based on the computational modelling of our work, the FORE-SIGHT NIRS device by CAS Medical source detector layout provides the greatest sensitivity at depth for the purposes of cortical monitoring in trauma. Variations in the layout have a significant impact on the quality of signal detected.
